# Legionella Pneumonia Associated With Rhabdomyolysis and Acute Renal Failure: A Case Report

**DOI:** 10.7759/cureus.107537

**Published:** 2026-04-22

**Authors:** David Kim, Soo Jeong Seo, Kirollos Gerges, Ravi Siriki

**Affiliations:** 1 Internal Medicine, NewYork-Presbyterian Queens, Flushing, USA; 2 Internal Medicine, Arkansas College of Osteopathic Medicine, Fort Smith, USA; 3 Nephrology, NewYork-Presbyterian Queens, Flushing, USA

**Keywords:** acute tubular necrosis (atn), legionella pneumonia, medical intensive care unit (micu), rhabdomyolysis causing acute kidney injury, rhabdomyolysis with acute renal failure

## Abstract

Legionnaires' disease is a severe form of pneumonia caused by the bacterium *Legionella pneumophila*. It comprises around 5% of all community-acquired pneumonia, but up to 20% of all severe community-acquired pneumonia. It often presents with extrapulmonary manifestations, including but not limited to myocarditis, hyponatremia, renal failure, and diarrhea, that have increased in incidence over the last few decades. It has a high hospitalization and mortality rate. The *Legionella *triad of pneumonia, rhabdomyolysis, and acute renal failure, although rare, has even higher mortality rates, especially in vulnerable population groups. Early recognition, diagnosis, and prompt management can help decrease mortality rates and can help reduce costs by preventing admissions to the intensive care unit. We present the case of a man in his 70s with many comorbidities including heart failure, coronary artery disease, atrial fibrillation, and chronic kidney disease who presented with altered mental status, cough, and fevers, developed the *Legionella *triad, and required care in the intensive care unit, subsequently needing dialysis as an outpatient due to lack of renal recovery as an inpatient, to highlight the importance of prompt multidisciplinary management to improve overall outcomes.

## Introduction

*Legionella *is a gram-negative bacterium commonly found in aquatic environments. It can become a health hazard when improperly cleaned water reservoirs cause them to multiply and infect individuals through aerosolization [1]. Legionnaires' disease refers to a severe form of pneumonia specifically caused by *Legionella pneumophila*. It can present with various clinical presentations, including extrapulmonary manifestations, including but not limited to myocarditis, hyponatremia, renal failure, and diarrhea, ranging from mild disease to severe life-threatening sequela, particularly in elderly or immunocompromised patients. In these subpopulations, *Legionella pneumophila *remains the second most common form of community-acquired pneumonia in the intensive care unit [2]. While mortality rates are high in these populations, mortality can reach up to 40% in a rare manifestation labeled the *Legionella *triad, which includes pneumonia, rhabdomyolysis, and acute kidney injury, often leading to renal failure [2].

Prompt treatment with appropriate antibiotics such as fluoroquinolones or macrolides within 24 hours has been shown to reduce mortality and reduce intensive care unit stays [3]. Therefore, early diagnosis is essential to improve patient outcomes. However, this can remain challenging with the lack of formal guidelines on who should receive testing. We aim to educate clinicians on the *Legionella *triad to encourage early multidisciplinary management to improve outcomes and raise awareness to guide an effort toward a standardized approach when faced with this disease process.

## Case presentation

**Figure 1:**
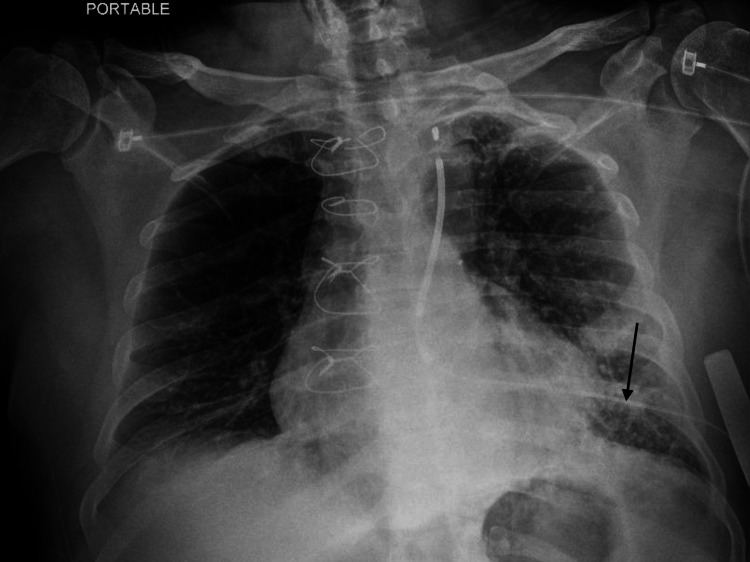
Chest radiograph depicting patchy left lower lung infiltrates (arrow)

Our case describes a man in his 70s with a past medical history of coronary artery disease s/p coronary artery bypass grafting (CABG), heart failure with reduced ejection fraction, atrial fibrillation, chronic kidney disease (CKD), and baseline serum creatinine (SCR) 2.5 mg/dL, who presented with confusion, cough, and fevers for a few days. On presentation, he was hypoxic, saturating 80-85% on room air, and required a high-flow nasal cannula of 40L/40% FiO2 to maintain saturations >90%. Initial chest radiograph showed patchy left lower lobe infiltrates (Figure 1).

Lab results were significant for mild leukocytosis, lactic acidosis, hyponatremia, and elevated SCR above his established baseline (Table 1). He was treated empirically with ceftriaxone and doxycycline upon admission for the management of his acute hypoxic respiratory failure (type I) secondary to community-acquired pneumonia.

**Table 1:** The patient's laboratory values showing the progression of values from day 1 of admission demonstrating the worsening renal injury and increased creatine kinase by day 3, indicative of rhabdomyolysis. The patient improved with appropriate antibiotics but ultimately required hemodialysis as an outpatient

	Laboratory values (day 1)	Laboratory values (day 3)	Laboratory values (day 10-discharge)	Reference ranges
White blood cell count (×10^3^/uL)	10.94	4.41	9.67	3.40-11.20
Sodium (mmol/L)	134	129	137	136-145
Potassium (mmol/L)	4.1	6.2	4.4	3.5-5.1
Creatinine (mg/dL)	3.68	9.36	7.79	0.67-1.17
Creatinine kinase (U/L)	N/A	15,150	440	20-200
Lactate (mmol/L)	2.9	N/A	0.85	0.50-1.60

Within three days of his hospital stay, his clinical status worsened with increasing oxygen requirements, requiring bilevel positive airway pressure (BiPaP) 40% FiO2 and 10 IPAP/5 EPAP settings, and worsening SCR with the presence of oliguria (<100 cc) (Table 1). Due to this rapid decline of renal function, the acute kidney injury workup was initiated. The renal bladder ultrasound was negative for hydronephrosis, but urinalysis sediment demonstrated granular casts and renal tubular cells, with elevated creatine kinase (CK) (Table 1). These findings were suggestive of the development of acute tubular necrosis (ATN) secondary to rhabdomyolysis. With increasing oxygen requirements, a history of congestive heart failure (CHF), and the presence of oliguria, he could not receive aggressive intravenous fluids due to the concern of developing fluid overload. He eventually tested positive for urine *Legionella *antigen; therefore, the antibiotic regimen was switched to azithromycin therapy. With the initiation of therapy, by days 4-5 of hospitalization, his respiratory status clinically improved, being weaned to nasal cannula oxygen therapy, but his SCR continued to worsen with anuria and electrolyte derangements, including hyperkalemia to >6.5. Therefore, hemodialysis was initiated.

With hemodialysis, he was weaned to room air. However, he showed minimal improvement in kidney function after several sessions of hemodialysis as an inpatient. He was arranged for hemodialysis in the outpatient setting due to a lack of renal recovery. One month later, he returned to the hospital for cardiac issues, and during the hospital stay, he showed signs of renal recovery with increased urine output and stable SCR without the need for hemodialysis.

## Discussion

Legionnaires' disease requires prompt diagnosis and treatment with the literature reporting on the delay of antibiotic treatment leading to increased frequency of intensive care unit admissions [3]. The literature has reported not only on the scarcity of *Legionella*-induced rhabdomyolysis and acute kidney injury but also on its importance due to the possibility of rapid clinical deterioration associated with a high mortality rate [4,5]. The *Legionella *triad, while uncommon, is associated with up to a 40% mortality rate and the need for renal replacement therapy in most patients [2]. This highlights the importance of being receptive to monitoring extrapulmonary manifestations to aid in early diagnosis to prevent complications and lower mortality rates. The range of reported extrapulmonary manifestations is wide and includes sepsis, cellulitis, soft tissue abscesses, septic arthritis, prosthetic joint infection, osteomyelitis, myocarditis, pericarditis, endocarditis, peritonitis, pyelonephritis, acute renal failure, rhabdomyolysis, dysarthria, and meningitis.

There are currently several modalities for diagnosis. A meta-analysis study completed in 2022 demonstrated that the sensitivity and specificity of urine antigen tests were moderate and high, respectively, indicating them as a useful tool for the early detection of *Legionella *[6]. However, older studies have offered conflicting viewpoints and argued that urinary antigen testing has poor sensitivity and specificity, with the need for clarification on future guidelines to determine which patient populations should receive testing [7]. One can argue that the patient populations, particularly susceptible to severe disease, such as the elderly or immunocompromised, should undergo testing, but different facilities often have different workflows. Different studies have employed different clinical diagnostic criteria to rule out *Legionella*. Haubitz et al. utilized six diagnostic criteria, namely, fever, cough, hyponatremia, elevated lactate dehydrogenase, inflammatory markers, and platelet count, with fewer than two criteria having a negative predictive value of 99% [8].

Current guidelines recommend either a fluoroquinolone, such as levofloxacin, or a macrolide, such as azithromycin, for first-line therapy [9]. Although clinical cure was comparable between the two groups, overall 30-day mortality was higher for macrolides. Subsequent subgroup analysis showed a decrease in hospital stay for levofloxacin, although mortality and adverse effects were comparable. The optimal duration has not been established, but generally depends on the severity of the disease, with most guidelines suggesting a 7-10-day course for severe disease [3]. We advise clinicians to be cognizant of extrapulmonary manifestations, as early diagnosis and appropriate treatment can potentially improve outcomes and lower mortality rates. We also highlight the need for standardized guidelines in testing, particularly for vulnerable populations, to aid in early diagnosis.

## Conclusions

This report acknowledges the high mortality rates of Legionnaires' disease in vulnerable population groups. We aim to educate the public on recognizing the symptoms of Legionnaires' disease to prompt early diagnosis and treatment with the goal of decreasing mortality rates and cutting costs by preventing admissions to the intensive care unit. There are currently no universal guidelines in place regarding *Legionella *testing, with facilities often having differing criteria for testing. Perhaps in the future, a universal diagnostic algorithm that is cost-effective can reach a consensus to improve overall mortality in vulnerable population groups. 
